# Energy metabolism in mobile, wild-sampled sharks inferred by plasma lipids

**DOI:** 10.1093/conphys/cox002

**Published:** 2017-02-14

**Authors:** Austin J. Gallagher, Rachel A. Skubel, Heidi R. Pethybridge, Neil Hammerschlag

**Affiliations:** 1 Rosenstiel School of Marine and Atmospheric Science, University of Miami, Miami, FL, USA; 2 Fish Ecology and Conservation Physiology Laboratory, Department of Biology and Institute of Environmental Science, Carleton University, Ottawa, ON, Canada; 3 Beneath the Waves, Inc., Miami, FL, USA; 4 Leonard and Jayne Abess Center for Ecosystem Science and Policy, University of Miami, Coral Gables, FL, USA; 5 CSIRO Oceans and Atmosphere Research, Hobart, Australia

**Keywords:** Cholesterol, fatty acid, metabolite, nutrition, shark, triglyceride

## Abstract

Evaluating how predators metabolize energy is increasingly useful for conservation physiology, as it can provide information on their current nutritional condition. However, obtaining metabolic information from mobile marine predators is inherently challenging owing to their relative rarity, cryptic nature and often wide-ranging underwater movements. Here, we investigate aspects of energy metabolism in four free-ranging shark species (*n* = 281; blacktip, bull, nurse, and tiger) by measuring three metabolic parameters [plasma triglycerides (TAG), free fatty acids (FFA) and cholesterol (CHOL)] via non-lethal biopsy sampling. Plasma TAG, FFA and total CHOL concentrations (in millimoles per litre) varied inter-specifically and with season, year, and shark length varied within a species. The TAG were highest in the plasma of less active species (nurse and tiger sharks), whereas FFA were highest among species with relatively high energetic demands (blacktip and bull sharks), and CHOL concentrations were highest in bull sharks. Although temporal patterns in all metabolites were varied among species, there appeared to be peaks in the spring and summer, with ratios of TAG/CHOL (a proxy for condition) in all species displaying a notable peak in summer. These results provide baseline information of energy metabolism in large sharks and are an important step in understanding how the metabolic parameters can be assessed through non-lethal sampling in the future. In particular, this study emphasizes the importance of accounting for intra-specific and temporal variability in sampling designs seeking to monitor the nutritional condition and metabolic responses of shark populations.

## Teaser

We evaluated concentrations of plasma lipids in four large, free-ranging shark species via non-lethal biopsies for the purposes of improving our understanding of their nutritional ecology and for assessing the utility of biopsies in future monitoring studies of the energetics and metabolism in shark populations.

## Introduction

Nutrition and energy metabolism in wild animals are both conceptualized and measured by a variety of methods, ranging from techniques that span behavioural approaches, morphological and developmental measurements and ecological interactions (Raubenheimer *et al*., 2009). The metabolic building blocks and pathways that animals use to obtain, transfer, store and use energy over time are fundamentally linked to their productivity and survival. Moreover, the demand for energy constrains the behaviour of animals (Speakman, 1997), which is fundamental to life-history variation and thus intrinsically connected to natural selection (Elliott *et al*., 2014). Yet, in the purest sense, nutrition and metabolism link individuals to the function of communities and structure of entire populations (Raubenheimer *et al*., 2009).

Lipids, including fatty acids and sterols, are a diverse group of metabolites (small molecule intermediates and products of metabolism) that play a critical role in almost all aspects of biological life. Lipids are under tight homeostatic control, but their composition, interaction and levels in organismal tissues are dynamic, which reflects long-term and health-related responses to changes in diet or other environmental variations (Orešič, 2009). In teleost and elasmobranch fishes, lipid-derived measurements have been used as metrics of nutritional condition, tissue damage and recent feeding events and to elucidate life histories (Norton *et al*., 2001; Wagner and Congleton, 2004; Yan *et al*., 2012). Three lipid classes play crucial roles in energy transport and are commonly used to study metabolism: triglycerides (TAG), free fatty acids (FFA) and total cholesterol (CHOL; Han, 2016; Orešič, 2009). Triglycerides (three fatty acids esterified to glycerol) are a main source of metabolic energy in many organisms and are thought to respond relatively quickly to changes in feeding. Free fatty acids (also known as non-esterified fatty acids) are also metabolic fuels; they are considered dynamic and the most metabolically active of the lipids. CHOL is an unsaturated alcohol of the steroid family and is essential for cell membrane production and a precursor for steroid hormones and further lipid transport. In most organisms, plasma transports these exogenous (dietary-derived) or endogenous (liver-produced) lipids towards different tissues, where they are stored or oxidized (Babin and Vernier, 1989). Strategies of lipid metabolism, including absorption and depositional processes, appear different between the different animal classes, reflecting distinct evolutionary paths (Speers-Roesch *et al*., 2006; Mazurie *et al*., 2010).

Sharks are a diverse group of marine predatory fishes. Many of the larger species undergo extensive migrations linked to foraging and reproduction (Hammerschlag *et al*., 2011; Domeier and Nasby-Lucas, 2013; Hussey *et al*., 2015; Graham *et al*., 2016), and are slow-growing, exhibiting long gestation periods that produce relatively few, highly developed offspring (Lucifora *et al*., 2002). Many species of sharks are threatened and experiencing population declines attributable to overfishing and habitat loss (e.g. Ferretti *et al*., 2010; Gallagher *et al*., 2012). Therefore, there is significant interest in enhancing an understanding of key physiological states that may help to inform the conservation and management of threatened populations (Simpfendorfer *et al*., 2011). However, measurements and temporal profiles of metabolites for most species of shark are not available.

Compared with teleost fish, the energy metabolism of sharks is considered unusual, being characterized by limited extrahepatic fatty acid oxidation capacity (Zammit and Newsholme, 1979; Ballantyne, 1997; Speers-Roesch and Treberg, 2010), albumin activation and utilization of lipoprotein, which bind to FFA (Metcalf and Gemmell, 2005). Experimental studies investigating the response of shark plasma lipids to diet have shown mixed results; dogfish (*Squalus* spp.) plasma lipids were shown not to be responsive to feeding to the same degree as in teleosts (Wood *et al*., 2010). After 150 days of starvation, dogfish FFA concentrations decreased, whereas ketone concentrations increased, suggesting their importance as fuel during food shortages (Zammit and Newsholme, 1979). The fatty acid profiles in the plasma of Port Jackson sharks (*Heterodontus portjacksonii*) have been shown to reflect short-term dietary changes (Beckmann *et al*., 2013a, b), which conforms to work showing that plasma lipids in other classes of organisms (birds, mammals and teleosts) vary proportionally to dietary composition (e.g. Grundy and Denke, 1990; Tocher, 2003; Käkelä *et al*., 2009). There is a clear opportunity to explore the overall usefulness of evaluating plasma lipid metabolites and dynamics in sharks, including an understanding of the extent of within- and between-species variation and how this may relate to different behavioural, genetic and environmental factors. Although undertaking experimental studies would be optimal, they are often not possible on large, mobile shark species of high conservation concern, demanding non-lethal ways of acquiring such information (Hammerschlag and Sulikowski, 2011).

Here, we present an exploratory and comparative study of three metabolite parameters (using non-lethal blood biopsy) in adult specimens of four species of free-ranging sharks from the subtropical Atlantic Ocean. As energetic requirements are driven by both intrinsic (biological) and extrinsic (environmental) factors, we also evaluate the significance of location, date, season, shark length and sex on the variability in measured metabolite levels. Specifically, we used these data to address the following questions. (i) What are the concentrations of plasma TAG, FFA and CHOL in the species assessed and how do they differ among individuals and between species? (2) How are these metabolic parameters related to one another? (3) Do concentrations of plasma TAG, FFA and CHOL differ greatly over time? We discuss our findings as they may relate to metabolic pathways and various biological traits (diet, habitat quality and life-history strategies) of marine predators.

## Materials and methods

### Sampling sites, species and capture

This study was conducted in four locations along a subtropical latitudinal gradient off Florida: off Miami and Soldier Key, Biscayne Bay, FL, USA (25.61°N, 80.17°W); the reef edge in the mid-Florida Keys National Marine Park in US federal waters (24.69°N, 80.85°W); inside Florida state waters within Everglades National Park (~25.0°N, 81.0°W); and off the west end of Grand Bahama Island, Bahamas (~26.6°N, 79.1°W). Sampling was conducted throughout the wet and dry seasons from 2011 to 2015, and tissue sampling efforts focused on the following four species of sharks: blacktip sharks (*Carcharhinus limbatus*), bull sharks (*Carcharhinus leucas*), nurse sharks (*Ginglyostoma cirratum*) and tiger sharks (*Galeocerdo cuvier*). All sharks were captured using circle-hook drumlines, a passive fishing technique (as described by Gallagher *et al*., 2014b). Each fishing unit consisted of a submerged weight base tied to a line running to the surface by means of an attached, inflatable buoy float. A 23 m monofilament gangion line (~400 kg test) was attached to the submerged weight by a swivel, which terminated at a baited 16/0, 5° offset circle hook. This method permitted any captured sharks to swim in a 23 m radius circle around the base. After 1 h, each drumline was sequentially checked for shark presence. Captured sharks were slowly brought to the boat and restrained on a partly submerged dive platform. Once landed, a water pump moving fresh seawater was inserted into the shark's mouth to facilitate respiration. This capture and handling method is used to promote shark vitality and minimize stress levels during sampling (Gallagher *et al*., 2014a). For each individual captured, sex was recorded and stretched total length (TL) was measured to the nearest centimetre over a straight line along the axis of the body.

### Haematological collection and metabolite analyses

Whole blood (~10 ml) was collected from the caudal vein using cooled 18 gauge needles and 10 ml syringes. Approximately 7 ml of the mixed whole blood samples was then centrifuged at 1300***g*** for 5 min to separate plasma. Samples were frozen on board, then transferred to a −20°C freezer on shore, where they were stored for future analyses. We ran three separate nutritional metabolic assays on the resulting plasma samples: triglycerides (in millimoles per litre; EnzyChrom Triglyceride Assay Kit, BioAssay Systems; Haywood, CA, USA), free fatty acids (in millimoles per litre; EnzyChrom Free Fatty Acid Assay Kit; BioAssay Systems) and total CHOL (in millimoles per litre; EnzyChromTM AF Cholesterol Assay Kit; BioAssay Systems), on a 96-well microplate absorbance reader at 570 nm (Tecan Sunrise, Tecan, Grödig, Austria); concentrations were determined using the appropriate standard curves. Although we performed each analysis for the majority of individuals in the present study, for certain individuals we did not run all metabolite analyses owing to blood plasma availability and collection anomalies (e.g. low volume). The ratio of TAG and CHOL was calculated as a potential index of nutritional condition (Amara and Galois, 2004; Giraldo *et al*., 2013; Sardenne *et al*., 2016) on the basis that in fishes TAG concentrations, indicative of energy reserves, are often positively correlated with body size, whereas CHOL concentrations are relatively stable over time.

### Statistical analyses

All data were logarithmically transformed to meet parametric assumptions. We compared mean values in all metabolites among species by using analysis of variance (ANOVA). To identify potential relationships among variability between metabolites and biological and environmental predictor variables, we first conducted principal component analysis (PCA) on the ranked metabolite and TL values, capture site and year, species and season (wet = June–September, dry = October–May). This analysis included only blacktip, bull and nurse sharks because we had only TAG values for tiger sharks (thus they were excluded from the PCA). A generalized linear model (GLM) was constructed to explore relationships between each metabolite and shark species, sex, animal TL, year, month and capture location (Biscayne Bay, outer reef, Everglades National Park or the Bahamas). Spearman correlations were used to evaluate patterns of metabolites, both within species and among all species combined. Statistical significance was declared at *P* < 0.05, and all analyses were conducted in MATLAB (Mathworks, Inc.) and R (R Core Team).

## Results

From May 2011 to May 2015 we captured, sampled and released a total of 281 sharks composed of 61 blacktip sharks, 45 bull sharks, 82 nurse sharks and 93 tiger sharks (Table 1).

**Table 1:** Mean total length, triglycerides, free fatty acids and cholesterol concentrations of all sampled sharks from the present study

	Total length (cm)			Triglycerides (mmol l^−l^)			Free fatty acids (mmol l^−l^)			Cholesterol (mmol l^−l^)		
Species	Mean	SD	*n*	Mean	SD	*n*	Mean	SD	*n*	Mean	SD	*n*
Blacktip	150.5	20.8	61	1.59	1.89	61	0.80	0.59	46	1.39	0.34	41
Bull	217.5	36.5	45	0.83	0.40	39	0.67	0.45	33	1.54	0.83	36
Nurse	223.2	34.9	82	1.66	1.65	80	0.13	0.19	42	0.99	0.39	38
Tiger	295.2	63.0	75	0.30	0.40	75	–	–	–	–	–	–

The largest species in our study was the tiger shark (293.0 ± 64.3 cm TL; Table 1), followed by the bull shark (217.5 ± 36.5 cm), blacktip shark (150.5 ± 20.8 cm) and nurse shark (223.2 ± 34.9 cm). The ranges of the sizes sampled here suggested that the majority of individuals captured were either adults or sub-adults; we did not encounter individual sizes indicative of early juveniles, young-of-year or neonates for any species (Table 1).

**Figure 1:**
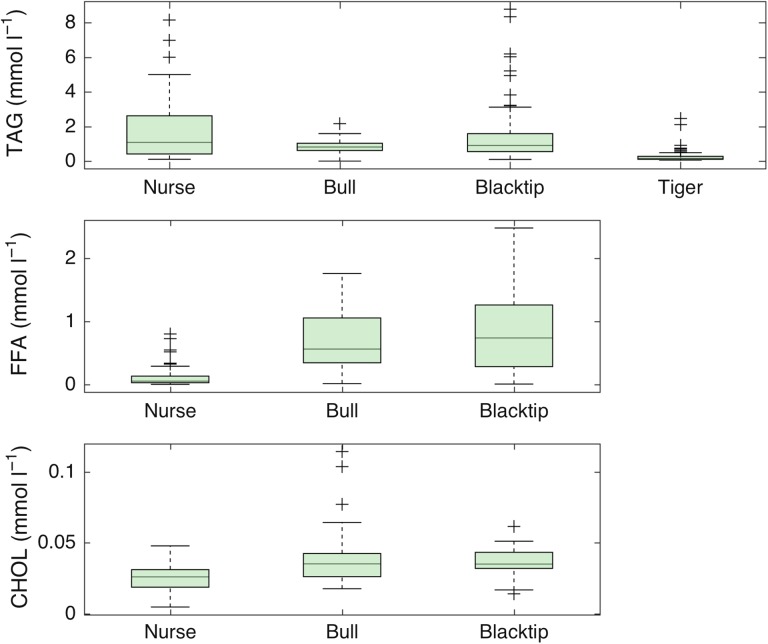
Triglyceride (TAG), free fatty acid (FFA) and cholesterol (CHOL) concentrations (in millimoles per litre) of nurse, bull, blacktip and tiger sharks over the study period from May 2011 to May 2015.

For all shark species combined, metabolite values ranged as follows (in millimoles per litre): TAG, 0.0031–8.79, 1.45 ± 1.60; FFA, 0.0031–2.48, 0.53 ± 0.53; and CHOL, 0.18–4.42, 1.30 ± 0.60. We detected a wide range of species-specific differences in metabolite values (Table 1 and Fig. 1). Nurse and blacktip sharks had the highest TAG values (1.66 ± 1.65 and 1.59 ± 1.89 mmol l^−1^, respectively; Table 1) followed by bull sharks (0.83 ± 0.40 mmol l^−1^), with tiger sharks having the lowest values (0.30 ± 0.40 mmol l^−1^; Table 1 and Fig. 1a). Blacktip sharks had the highest FFA concentrations (0.80 ± 0.59 mmol l^−1^), followed by bull and then nurse sharks (0.67 ± 0.45 and 0.13 ± 0.19 mmol l^−1^, respectively; Table 1 and Fig. 1b). Likewise, bull and blacktip sharks had the greatest CHOL values (1.54 ± 0.83 and 1.39 ± 0.34 mmol l^−1^, respectively; Table 1 and Fig. 1c) and nurse sharks the lowest (0.99 ± 0.39 mmol l^−1^). These differences were species specific (ANOVA; TAG, *F*_4,253_ = 47.65, *P* < 0.0001; FFA, *F*_3,119_ = 45.97, *P* < 0.0001; and CHOL, *F*_3,113_ = 13.59, *P* < 0.0001; Table 2). Ratios of TAG/CHOL, used as an additional nutritional proxy, were of a similar range for nurse and blacktip sharks (2.01 ± 3.17 and 1.62 ± 1.63, respectively), which were higher than those found in bull sharks (0.67 ± 0.44).

**Table 2:** *P*-values of an analysis of variance comparing means among all species using logarithmically transformed metabolite values

	Triglycerides	Free fatty acids	Cholesterol
Nurse vs. bull	0.14	**9.71E−10**	**3.50E−05**
Nurse vs. blacktip	1.00	**9.56E−10**	**6.04E−05**
Blacktip vs. bull	0.21	0.94	0.96
Tiger vs. nurse	**3.77E−09**	–	–
Tiger vs. bull	**4.08E−09**	–	–
Tiger vs. blacktip	**3.77E−09**	–	–

Bold denotes statistical significance (*P* < 0.05).

Principal components analysis yielded two distinct principal components (PCs), which collectively explained 65.8% of variability in the data set (Table 3). In the PC1, the predictors which explained most of the data variance were season, species, TL and year of capture (Table 3), whereas TL, year and species had similarly dominant eigenvectors in the PC2 (Table 3). Metabolite values were less influential on the data set's variability than the explanatory factors included.

**Table 3:** Principal eigenvectors from principal components analysis of metabolite values and explanatory variables for nurse, bull and blacktip sharks

	PC1	PC2
Species	−0.46	−0.45
Sex	0.32	−0.22
Length	−0.44	0.60
Season	0.53	−0.23
Year	−0.43	−0.57
Site	−0.11	0.04
Triglycerides	−0.0095	0.10
Free fatty Acids	0.077	0.042
Cholesterol	0.051	−0.0042

**Figure 2:**
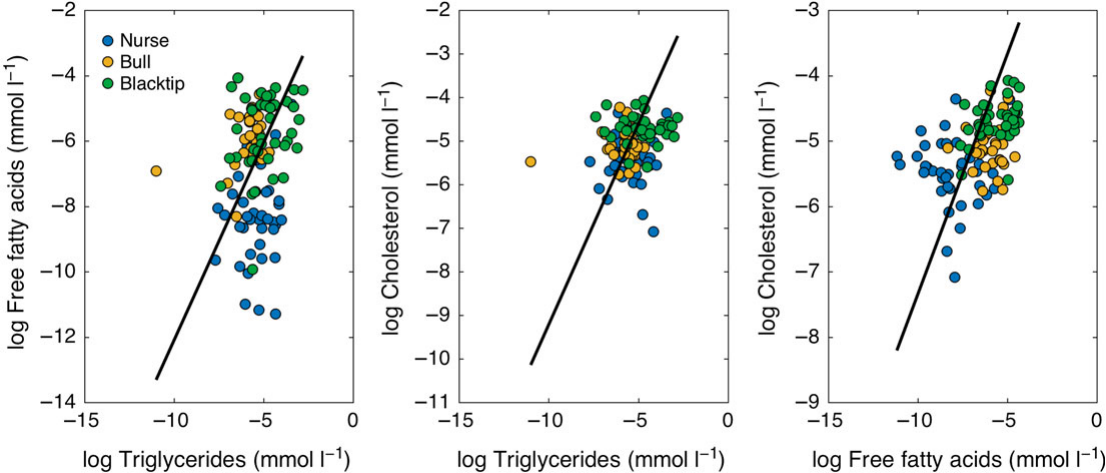
Relationships of logarithmically transformed metabolites triglycerides, free fatty acids and cholesterol for nurse, bull and blacktip sharks.

Species effects were significant in the GLMs for all of the three metabolites (*P* < 0.0001; Table 4). Shark TL was the only other significant variable in the model for CHOL, whereas year and month of capture were also significant in the model for TAG (Table 4). Given the impact of TL on variability in the data set as illustrated by PCA (Table 4), we normalized metabolite values by TL before running pairwise comparisons. Each of the metabolites showed significant positive relationships with one another for all shark species combined, explaining between ~20 and 50% of the variability (Fig. 2).

**Table 4:** *P*-values of a generalized linear model for logarithmically transformed metabolite values

	Triglycerides	Free fatty acids	Cholesterol
Species	**2.18E−04**	**1.91E−09**	**7.93E−07**
Location	0.066	0.47	0.48
Year	**0.048**	0.62	0.38
Month	**0.016**	0.22	0.56
Length	0.52	0.57	**7.87E−04**
Sex	0.57	0.79	0.20

Location refers to shark capture location (Biscayne Bay, Everglades, Florida Keys or the Bahamas). Bold denotes statistical significance (*P* < 0.05).

**Figure 3:**
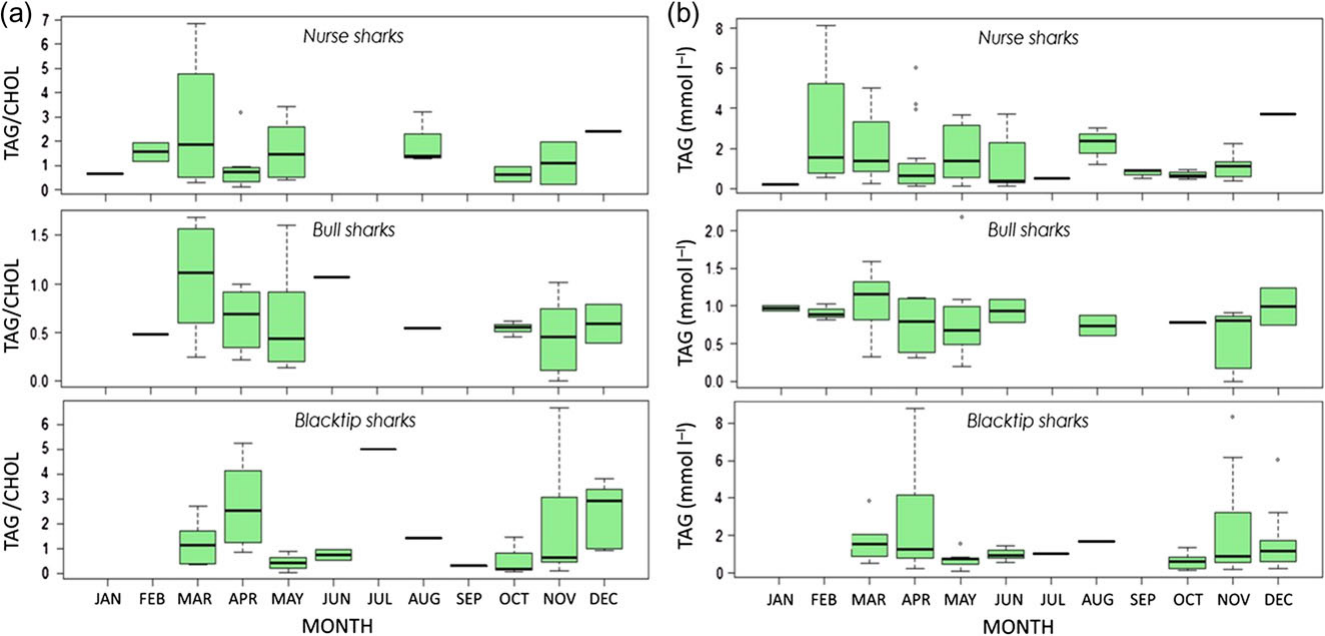
Inter-annual variability of the ratio of triglyceride to cholesterol (TAG/CHOL; **a**) and triglyceride (TAG; in millimoles per litre) on its own (**b**) for nurse, bull and blacktip sharks over the study period. Months are denoted on the *x*-axis.

**Figure 4:**
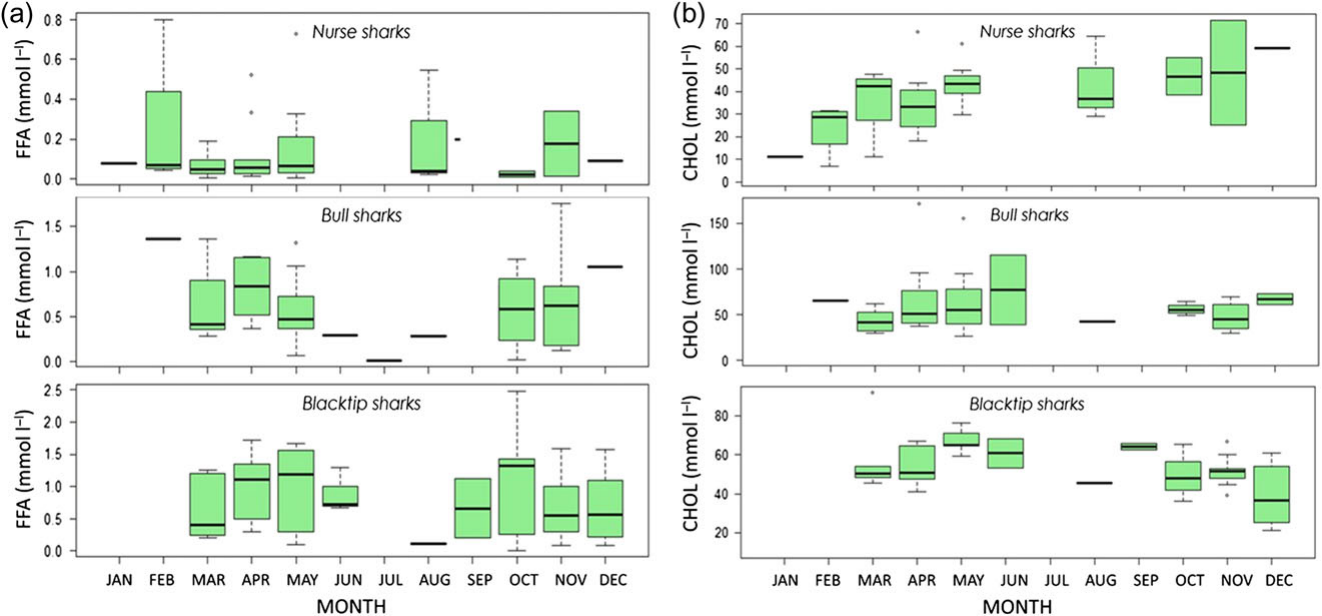
Inter-annual variability of free fatty acids (FFA, in millimoles per litre; **a**) and cholesterol (CHOL, in millimoles per litre; **b**) for nurse, bull and blacktip sharks over the study period. Months are denoted on the *x*-axis.

Intra-annual variability in some of the metabolites was detected in all species (Figs 3 and 4). In blacktip sharks, relative levels of TAG peaked in April and November (Fig. 3a), while FFA and CHOL remained relatively constant throughout the year (Fig. 4a and b). In nurse sharks, TAG was highest in February–April (Fig. 3a), while FFA and CHOL were highly variable throughout the year (Fig. 4a and b). In bull sharks, relative levels of TAG peaked in March (Fig. 3a), CHOL peaked in April–May (Fig. 4a), and FFA declined in June–August (Fig. 4a). Temporal trends in TAG/CHOL ratios followed that of TAG in all species (Fig. 3b).

## Discussion

Our investigation into plasma lipid dynamics in four free-ranging shark species provided baseline data on their natural variability in three metabolic parameters (TAG, FFA and CHOL) and insights into how these lipid metabolites may be used in studies monitoring shark nutritional health status over space and time. In our study, biological attributes (species and body size) and environmental features (season and year) had the greatest influence on variability in the plasma lipid spectra. The importance of these factors to our data set highlights the relationship between animal physiology and life history (Ricklefs and Wikelski, 2002), and the complex and unusual dynamics of lipid metabolism in sharks (Valls *et al*., 2016).

Plasma TAG, FFA and CHOL were positively related across species, highlighting a common organization of lipid metabolism among these taxa, as expected (Speers-Roesch and Treberg, 2010). However, a wide range of values among the three plasma metabolites were detected, and species effects loaded strongly in both components of our PCA, which may be related to the different ecologies, physiologies and behaviours of these sharks. Highly active sharks, such as blacktip and bull sharks, revealed higher concentrations of plasma FFA, whereas they were low in nurse sharks, a species known to be relatively sedentary, with low metabolic rates (Carrier and Pratt, 1998; Whitney *et al*., 2016). During non-migratory periods, blacktip and bull sharks have been shown to exhibit relatively restricted home ranges and high site fidelity to coastal areas (Papastamatiou *et al*., 2010; Hammerschlag *et al*., 2012; Graham *et al*., 2016). Sharks that occupy this ecological niche may meet their energetic requirements within their relatively small home ranges (Papastamatiou *et al*., 2009). These inter-species differences may also be explained by the fact that the larger sharks (bull and tiger), which were found in our study to have low FFA, have a lower mass-specific metabolic rate than that of smaller sharks and probably do not require as much energy to fuel movement (Carlson *et al*., 2004). Free fatty acids typically function as a transport form of lipids from storage depots to other tissues (i.e. muscle), meaning that highly mobile predators may have a large demand for FFA. In teleosts, FFA are considered the most metabolically active lipid fraction; however, FFA oxidation in sharks is considered to be more limited (Zammit and Newsholme, 1979; Ballantyne, 1997; Speers-Roesch and Treberg, 2010), with only recent work suggesting that FFA in shark plasma does reflect an exogenous pathway (i.e. recent feeding; Beckmann *et al*., 2013a, b). As such, we cannot pinpoint the specific pathway leading to FFA detection in the plasma of the study sharks. Our plasma FFA results are among the highest yet to be reported for an elasmobranch (Ballantyne *et al*., 1993; Speers-Roesch and Treberg, 2010), but are much lower than that typically reported in teleosts (e.g. seabass *Dicentrarchus labrax*, 2.22 ± 0.32 mmol l^−1^ and seabream *Chrysophyrys auratus*, 2.79 ± 0.62 mmol l^−1^; McClelland *et al*., 1995), which is in agreement with earlier work showing that teleosts have 10-fold higher concentrations of plasma FFA than sharks (Zammit and Newsholme, 1979).

Nurse sharks, which are relatively sedentary animals with a low metabolic rate and high cost of movement (Whitney *et al*., 2016), showed significantly higher concentrations of plasma TAG than other species assessed. It is thus possible that the mobilization of TAG may be more important for low-cost movements in sharks. The two study species (tiger and bull shark) that usually have the most generalist diet within the group of species investigated (Cortés, 1999) had the lowest TAG. High concentrations of TAG are found in the livers of many species of shark (Lipshaw *et al*., 1972; Pethybridge *et al*., 2011; Beckmann *et al*., 2013a, b), and TAG is typically bound to lipoproteins when circulating in the blood (Mills *et al*., 1977). It is possible that the low TAG concentrations found here could be attributed to the consumption of a broad diet of both high- and low-quality nutritional items. Plasma TAG concentrations have been linked to body condition, as Gallagher *et al*. (2014a) found a significant relationship between plasma TAG and body condition in tiger sharks, with girthier animals (relative to length) having higher energy stores. Yet, other work has starved smaller sharks and not found a link between TAG and condition (Zammit and Newsholme, 1979). The high variability in TAG may also reflect recent feeding activity, different physiological states (maturation, fasting, resting, temperature, etc.) or even genetic variability. Additional research is needed to investigate these potential mechanisms further.

Sharks are considered to have a low capacity for the transport of CHOL (Larsson *et al*., 1976; Larsson and Fange, 1977). Thus, the detection of cholesterol in the plasma could be used as an indicator of tissue catabolism, although it is clearly also found in the diet. The highest CHOL values in our study were evident in bull sharks, and our values are similar to those reported in blue sharks (*Prionace glauca*) and mako sharks (*Isurus oxyrinchus*; Stillwell and Kohler, 1982). In our study, CHOL was significantly affected by shark size (Table 4), indicating that as sharks (particularly the bull shark) increase in size they may have greater access (i.e. gape and experience) to larger, more profitable prey, such as turtles and marine mammals (both groups inhabit our sampling locations; Hart and Fujisaki, 2010; Litz *et al*., 2012). We did not measure CHOL concentrations in any prey species, however, and future work should seek to compare lipid profiles of prey items with those measured in predators. There were large differences in the ratios of TAG/CHOL observed between sharks, suggesting that bull sharks have a lower nutritional condition than both blacktip and nurse sharks that shared similar value ranges. Although we cannot pinpoint the reason for this result, it may be related to foraging strategies, physiological differences or other biotic factors that we did not measure (i.e. reproduction). We do not know whether our standardized capture methods affected the metabolite values in sharks (although they were likely to affect them in a similar manner); however, our fishing technique included short fishing times (<1 h) and allowed the sharks to swim in circles when hooked, thus permitting respiration (Gallagher *et al*., 2014b).

The finding that each of the metabolites was significantly correlated with one another (Table 5 and Fig. 2) may indicate that our physiological parameters can be used to trace differences or trade-offs in food availability and activity in sharks over space and time. This concept is supported by the observed significant effects of year and month in TAG results and temporal variability in all metabolites (Figs 3 and 4). Plasma TAG concentrations and TAG/CHOL ratios were found to be relatively constant during the year but peaked in February–April for blacktip, bull and nurse sharks (Fig. 3). This temporal shift in TAG may correspond to greater ecosystem productivity and a pulse in food availability during this period (Lirman *et al*., 2008), as the concentrations appeared to drop off during the warmer summer months. The agreement between intra-annual patterns in TAG concentrations and TAG/CHOL suggests that TAG is an important metabolite for the condition of sharks over time. Relatively constant concentrations of FFA and CHOL (as documented here; Fig. 4), interspersed with peaks at discrete times throughout the year (e.g. bull sharks; Fig. 4b) may be linked to balanced energy metabolism interspersed with sporadic feeding pulses/episodes used to support movements requiring large amounts of energy. Seasonal variations in plasma CHOL concentrations in species of fish have been linked to spawning (Larsson and Fange, 1977). Constant concentrations of FFA and changing TAG may suggest a concurrent utilization of endogenous and exogenous energy sources where metabolic costs are balanced, and future work should expand on these temporal patterns. Clearly, more sampling is needed in the summer months (Table 6), especially as shark occurrence is generally lower in our sampling regions during this time (our unpublished data). However, the bi-modal patterns detected in the metabolite values for some species here provide an important link between behaviour (residency, movements) and physiology (energy) that could be especially valuable for understanding the mechanisms of decision-making in sharks.

**Table 5:** Spearman correlation coefficients of relationships for length-normalized metabolites and the slope of the relationship

	Slope	*r*	*P*-value
TAG vs. FFA	0.21	0.27	**0.036**
TAG vs. CHOL	0.36	0.23	**0.017**
FFA vs. CHOL	1.30	0.52	**4.70E−09**

Abbreviations: CHOL, cholesterol; FFA, free fatty acid; and TAG, triglyceride. Bold denotes statistical significance (*P* < 0.05).

**Table 6:** Intra-annual sampling coverage (*n* values, by month) in TAG, FFA, CHOL and TAG/CHOL replicates for blacktip, bull and nurse sharks in the present study

	Blacktip				Bull				Nurse			
	TAG	FFA	CHOL	T/C	TAG	FFA	CHOL	T/C	TAG	FFA	CHOL	T/C
J	0	0	0	0	1	0	0	0	1	1	1	1
F	0	0	0	0	3	1	1	1	7	4	3	2
M	6	5	5	5	6	5	6	6	16	8	6	6
A	8	5	5	5	6	6	6	6	18	10	9	9
M	7	5	5	3	9	9	8	8	13	11	10	10
J	7	3	2	2	2	1	1	1	8	0	0	0
J	0	0	0	0	0	1	0	0	0	0	0	0
A	1	1	1	1	2	1	1	1	4	3	3	3
S	2	2	2	2	0	0	0	0	2	0	0	0
O	7	5	3	3	3	4	3	3	3	2	2	2
N	13	12	12	12	5	5	5	5	7	2	2	2
D	10	7	5	5	2	1	2	2	1	1	1	1

Abbreviations: TAG, triglyceride; FFA, free fatty acid; CHOL, cholesterol; and T/C, ration of triglycerides to cholesterol.

### Conclusions

We investigated aspects of the nutrition and energy metabolism of multiple large, mobile shark species that co-occur over space and time using non-lethal biopsy sampling. As many of the specific dynamics of lipid metabolism and mobilization are still not well understood in large sharks, we affirm that this study is a needed preliminary step to understanding these parameters in the blood of free-ranging sharks, and these parameters could be candidate markers for understanding energy and nutrition in wild sharks. The range of values between and among species for each of the metabolites investigated here suggests that possible ecological (dietary), biological (shark size, metabolic rate, reproductive state) and environmental factors (time of year, season) may be important in explaining nutritional and energetic variation. However, we must be transparent that these inferences are in areas speculative and require further validation across species and time scales. The inter-species variation in metabolites documented here provides insight, but the underlying mechanisms that explain this diversity need exploring. We realize that we touched upon only a small subset of what a species’ ‘nutritional ecology’ entails, and the integration of additional nutritional parameters with behavioural and reproductive information may be particularly informative for future studies. Additionally, inclusion of other metabolites (i.e. ketone bodies; Valls *et al*., 2016) will help to improve our understanding of the dynamics and relative importance and/or contribution of these parameters to the nutritional ecology of shark species. Such information can ultimately improve our understanding of the mechanistic basis of animal movement, decision-making and even the health of entire habitats and prey sources, therefore helping us better link nutrition and health to the conservation physiology of sharks.
